# *Plantago ovata* F. Mucilage-Alginate Mucoadhesive Beads for Controlled Release of Glibenclamide: Development, Optimization, and *In Vitro-In Vivo* Evaluation

**DOI:** 10.1155/2013/151035

**Published:** 2013-04-04

**Authors:** Amit Kumar Nayak, Dilipkumar Pal, Kousik Santra

**Affiliations:** ^1^Department of Pharmaceutics, Seemanta Institute of Pharmaceutical Sciences, Jharpokharia, Mayurbhanj, Odisha 757086, India; ^2^Department of Pharmaceutical Sciences, Guru Ghasidas Vishwavidyalaya (A Central University), Koni, Bilashpur, Chhattisgarh 495 009, India

## Abstract

The current study deals with the development and optimization of ispaghula (*Plantago ovata* F.) husk mucilage- (IHM-) alginate mucoadhesive beads containing glibenclamide by ionotropic gelation technique. The effects of sodium alginate (SA) to IHM and cross-linker (CaCl_2_) concentration on the drug encapsulation efficiency (DEE, %), as well as cumulative drug release after 10 hours (R_10 h_, %), were optimized using 3^2^ factorial design based on response surface methodology. The observed responses were coincided well with the predicted values by the experimental design. The optimized mucoadhesive beads exhibited 94.43 ± 4.80% w/w of DEE and good mucoadhesivity with the biological membrane in wash-off test and sustained drug release profile over 10 hours. The beads were also characterized by SEM and FTIR analyses. The *in vitro* drug release from these beads was followed by controlled release (zero-order) pattern with super case-II transport mechanism. The optimized glibenclamide-loaded IHM-alginate mucoadhesive beads showed significant antidiabetic effect in alloxan-induced diabetic rats over prolonged period after oral administration.

## 1. Introduction

The mucoadhesive polymer containing oral drug delivery systems has the capacity to prolong residence time of drugs at the absorption site and facilitate intimate contact with underlying absorptive surface to enhance bioavailability [1]. Polymers used in the mucoadhesive formulations include natural, semisynthetic, and synthetic ones.

In recent years, a growing interest has been identified in the development of natural polymer-based drug delivery systems due to their biodegradability, biocompatibility, aqueous solubility, swelling ability, easy availability, and cost-effectiveness [2]. Amongst various natural polymers, alginates have been widely used in the development of drug delivery applications [3–6]. It is obtained from *Laminaria hyperborean, Ascophullum nodosum, *and *Macrocystis pyrifera *[1]. It is composed of linear copolymers of two monomeric units, that is, *β*-D-mannuronic acid and *α*-L-guluronic acid [7]. Sodium alginate (SA) undergoes ionotropic-gelation by Ca^2+^ to form calcium alginate due to an ionic interaction between carboxylic acid groups of alginate chain and Ca^2+^ [8]. Sodium alginate has mucoadhesive property; however, the cross-linked alginates are usually fragile [9, 10]. Therefore, blending of different mucoadhesive polymers is one of the most popular approaches to formulate ionotropically cross-linked alginate-based mucoadhesive beads [9, 11, 12]. Again, blending with suitable polymers, may improve the drug encapsulation, which is found comparatively lower in alginate-based beads prepared by ionotropic-gelation method [6].

Ispaghula (*Plantago ovata* F.) husk is an indigenous product of south Asia and is widely used herbal product both in traditional and modern medicines [13]. The ispaghula husk mucilage (IHM) is white, hydrophilic in nature, and forms a colorless gel in presence of water [14–16]. IHM contains a high amount of highly branched neutral arabinoxylan (arabinose 22.6%, xylose 74.6%, and traces of other sugars) and about 35% of nonreducing terminal sugar residues [16]. Few investigations had been carried out to formulate mucoadhesive beads as drug delivery matrices using both untreated ispaghula husk and alkaline treated ispaghula husk directly as polymeric blend with sodium alginate [1, 17, 18]. However, no attempt has been taken to formulate mucoadhesive alginate-based beads using isolated IHM as polymeric blend for the use in drug delivery. In the current investigation, the utility of isolated IHM, as a possible natural polymeric-blend with SA for the development of new IHM-blended alginate beads containing glibenclamide through ionotropic-gelation, was examined. 

Glibenclamide is a sulfonylurea used in the treatment of non-insulin-dependent diabetes mellitus (NIDDM, type-II) [2, 19]. Its plasma half-life is 4–6 hours, which makes multiple dosing to maintain the therapeutic blood level [2]. Therefore, it would be beneficial to develop a mucoadhesive system of glibenclamide using IHM-alginate for oral use, which might facilitate an intimate contact with the mucous membranes (i.e., mucoadhesion or bioadhesion) in the gastrointestinal tract, and thus the gastric residence could be prolonged to release glibenclamide at the target site at controlled rate over an extended period to maximize the therapeutic effect. 

In the development of any pharmaceutical formulation, an important issue is to design a formulation with optimized quality in a short time period and minimum number of trials [20, 21]. Traditionally, pharmaceutical formulators develop various formulations by changing one variable at a time while keeping others fixed. This classical method is laborious and time consuming. However, many experiments do not succeed in their purpose because they are not properly thought out and designed, and even the best data analysis cannot compensate lack of planning. Therefore, it is essential to understand the influence of formulation variables on the quality of formulations with a minimal number of experimental trials and subsequent selection of formulation variables to develop an optimized formulation using established statistical tools [5, 22–24]. Factorial designs, where all the factors are studied in all possible combinations, are considered the most efficient in estimating the influence of individual variables and their interactions performing minimum numbers of experiments [25]. A computer-aided optimization technique based on 3^2^ (two factors and three levels) factorial design and response surface methodology was employed to investigate the effects of two independent process variables (factors), that is, SA: IHM and cross-linker (CaCl_2_) concentration on the properties of glibenclamide-loaded ionotropically gelled IHM-alginate beads such as drug encapsulation and drug release.

## 2. Experimental

### 2.1. Materials

Glibenclamide (B.S. Traders Pvt. Ltd., India), SA (Central Drug House, India), ispaghula husk (Shree Baidyanath Ayurved Bhawan Pvt. Ltd., India), and calcium chloride (Merck Ltd., India) were used for this investigation. All other chemicals and reagents used were of analytical grade.

### 2.2. Isolation IHM from Ispaghula Husk

The mucilage from ispaghula (*Plantago ovata* F.) husk was extracted according to the previously reported method [26, 27]. An amount of 10 mL of 0.1 M HCl was heated to boil in a 100 mL flask and 1 gram of *Plantago ovata* F. husk was added. Heating was resumed and the process of husk dissolution was watched. After total change of color, the flask was finally removed from heat and the solution was filtered through a clean muslin cloth while still hot. In order to separate residual traces of mucilage, the seeds were washed twice with 5 mL of hot water and the solution obtained each time was filtered. The combined filtrate, containing the dissolved mucilage, was mixed with 60 mL of 95% ethyl alcohol, stirred, and allowed to stand for 5 hours. Finally, the supernatant liquid was decanted and the precipitate in the beaker was dried in an oven at 50°C. The dried IHM cake was grounded with a mortar and passed through a sieve (0.15 mm mesh size). The isolated IHM powders were packed in a plastic bag and kept airtight desiccators until further use.

### 2.3. Preparation of Glibenclamide-Loaded IHM-Alginate Beads

The glibenclamide-loaded IHM-alginate beads were prepared by ionotropic-gelation technique using calcium chloride (CaCl_2_) as cross-linker. Briefly, SA and IHM aqueous dispersions were prepared separately using distilled water. These dispersions were well mixed with stirring for 15 min at 1000 rpm using a magnetic stirrer (Remi Motors, India). Afterwards, glibenclamide was added to the dispersion mixture. The ratio of drug to polymer was maintained 1 : 2 in all formulations. The final mixtures were homogenized for 15 min at 1000 rpm using a homogenizer (Remi Motors, India) and ultrasonicated for 5 minutes for debubbling. The resulting dispersion was then added via a 21-gauge needle. The added droplets were retained in the CaCl_2_ solution for 15 min to complete the curing reaction and to produce rigid beads. The wet beads were collected by decantation and washed two times with distilled water and dried at 37°C for 24 h. The dried glibenclamide-loaded IHM-alginate beads were stored in a desiccator until used.

### 2.4. Experimental Design for Optimization

 A 3^2^ factorial design was employed for optimization with SA: IHM (*X*_1_) and concentration of CaCl_2_ (*X*_2_) as the prime selected independent variables, which were varied at three levels, low (–1), medium (0), and high (+1). The drug encapsulation efficiency (DEE, %) and 10 hours (*R*_10 h_, %) were used as dependent variables (responses). Design-Expert Version 8.0.6.1 software (Stat-Ease Inc., USA) was used for the generation and evaluation of the statistical experimental design. The matrix of the design including investigated responses, that is, DEE and *R*_10 h_, is shown in Table 1. The effects of independent variables upon the all measured responses were modelled using the following quadratic mathematical model generated by 3^2^ factorial design [5]:

(1) Y=b0+b1X1+b2X2+b3X1X2+b4X12+b5X22,

where *Y* is the response, *b*_0_ is the intercept, and *b*_1_, *b*_2_, *b*_3_, *b*_4_, *b*_5_ are regression coefficients. *X*_1_ and *X*_2_ are individual effects; *X*_1_^2^ and *X*_2_^2^ are quadratic effects; X_1_X_2_ is the interaction effect. One-way ANOVA was applied to estimate the significance (*P* < 0.05) of generated models. Individual response parameters were evaluated using the *F*-test. The response surface methodology was applied to analyze the effect of independent factors (SA: IHM and CaCl_2_ concentration) on the measured responses (DEE and *R*_10 h_).

### 2.5. Determination of DEE (%)

 Accurately weighed, 100 mg of beads were taken and were crushed using pestle and mortar. The crushed powders of drug containing beads were placed in 500 mL of phosphate buffer, pH 7.4, and kept for 24 hours with occasionally shaking at 37 ± 0.5°C. After the stipulated time, the mixture was stirred at 500 rpm for 15 min on a magnetic stirrer. The polymer debris formed after disintegration of bead was removed filtering through Whatman filter paper (No. 40). The drug content in the filtrate was determined using a UV-VIS spectrophotometer (Shimadzu, Japan) at 228.5 nm. The DEE of beads was calculated using this following formula:

(2) DEE  (%)=Actual  drug  content  in  beadsTheoretical  drug  content  in  beads×100.

### 2.6. Bead Size Measurement

 Particle size of 100 dried beads from each batch was measured by optical microscopic method for average particle size using an optical microscope (Olympus). The ocular micrometer was previously calibrated by stage micrometer.

### 2.7. Surface Morphology Analysis by Scanning Electron Microscopy (SEM)

 Samples were gold coated by mounted on a brass stub using double-sided adhesive tape and under vacuum in an ion sputter with a thin layer of gold (3 ~ 5 nm) for 75 seconds and at 20 kV to make them electrically conductive, and their morphology was examined by scanning electron microscope (ZEISS EVO 40, Japan).

### 2.8. Fourier Transform-Infrared (FTIR) Spectroscopy

Samples were reduced to powder and analyzed as KBr pellets by using a Fourier transform-infrared (FTIR) spectroscope (Perkin Elmer Spectrum RX I, USA). The pellet was placed in the sample holder. Spectral scanning was taken in the wavelength region between 4000 and 400 cm^−1^ at a resolution of 4 cm^−1^ with scan speed of 1 cm/second.

### 2.9. *In Vitro* Drug Release Studies

 The release of glibenclamide from various IHM-alginate beads was tested using a dissolution apparatus USP (Campbell Electronics, India). The baskets were covered with 100-mesh nylon cloth to prevent the escape of the beads. The dissolution rates were measured at 37 ± 1°C under 50 rpm speed. Glibenclamide-loaded IHM-alginate beads equivalent to 30 mg glibenclamide were taken in 900 mL of dissolution medium (0.1 N HCl, pH 1.2 for first 2 hours and phosphate buffer, pH 7.4 for next 8 hours). An amount of 5 mL of aliquots was collected at regular time intervals, and the same amount of fresh dissolution medium was replaced into the dissolution vessel to maintain sink condition throughout the experiment. The collected aliquots were filtered and suitably diluted to determine absorbance using a UV-VIS spectrophotometer (Shimadzu, Japan) at 228.5 nm against appropriate blank.

In order to predict and correlate the *in vitro* release behaviour of glibenclamide from formulated glibenclamide-loaded IHM-alginate beads, it is necessary to fit into a suitable mathematical model. The *in vitro *drug release data were evaluated kinetically in different mathematical models [5, 6]:

(i) *Zero-Order Model: Q* = *kt* + *Q*_0_, where *Q* represents the drug released amount in time *t*, and *Q*_0_ is the start value of *Q*; *k* is the rate constant;
(ii) *First-Order Model: Q* = *Q*_0_ *e* ^*kt*^, where *Q* represents the drug released amount in time *t*, and *Q*_0_ is the start value of *Q*; *k* is the rate constant;
(iii) *Higuchi Model: Q* = *kt* ^0.5^, where *Q* represents the drug released amount in time *t*, and *k* is the rate constant;
(iv) *Korsmeyer-Peppas Model: Q* = *kt* ^*n*^, where *Q* represents the drug released amount in time *t*, *k* is the rate constant, and *n* is the diffusional exponent, indicative of drug release mechanism.

The Korsmeyer-Peppas model was also employed in the *in vitro *drug release behaviour analysis of these formulations to distinguish between competing release mechanisms [5, 6]: Fickian release (diffusion-controlled release), non-Fickian release (anomalous transport), and case-II transport (relaxation-controlled release). When *n* is ≤0.43, it is Fickian release. The *n* value between 0.43 and 0.85 is defined as non-Fickian release. When *n* ≥ 0.85, it is case-II transport.

### 2.10. Mucoadhesion Testing

The mucoadhesivity of optimized glibenclamide-loaded IHM-alginate beads was evaluated by *ex vivo *wash-off method [11, 12]. Freshly excised pieces of goat intestinal mucosa (2 × 2 cm) (collected from slaughterhouse) were mounted on glass slide (7.5 × 2.5 cm) using thread. Fifty beads were spread onto the wet tissue specimen, and the prepared slide was hung onto a groove of disintegration test apparatus. The tissue specimen was given regular up and down movement in a vessel containing 900 mL of 0.1 N HCl (pH 1.2) and phosphate buffer (pH 7.4), separately, at 37°C. After regular time intervals, the machine was stopped and the number of beads still adhering to the tissue was counted.

### 2.11. *In Vivo* Evaluation

*In vivo *studies were performed in alloxan-induced diabetic albino rats [12] of either sex (weighing 266–342 grams). The acclimatized rats were kept fasting for 24 h with water *ad libitum*. All experiments were performed between 8 AM to 12 PM to minimize circadian influences. The experimental protocol was subjected to the scrutiny of the Institutional Animal Ethical Committee and was cleared before starting. The animals were handled as per guidelines of committee for the purpose of control and supervision on experimental animals (CPCSEA). All efforts were made to minimize both the suffering and number of animals used. 

 The rats were made diabetic by intraperitoneal administration of freshly prepared alloxan solution at a dose of 150 mg/kg dissolved in 2 mM citrate buffer (pH 3.0). After one week of alloxan administration, alloxanized rats with fasting blood glucose of 300 mg/dL or more were considered diabetic and were employed in the study for 12 hours. The alloxan-induced diabetic rats were divided randomly into 2 groups of 6 rats each and treated as follows. Group A was administered with pure glibenclamide in suspension form, and Group B was administered with optimized formulation of glibenclamide-loaded IHM-alginate beads, both at a dose equivalent to 5 mg glibenclamide/kg body weight using oral feeding needle. Blood samples were withdrawn (0.1 mL) from tail tip of each rat at regular time intervals under mild ether anesthesia and were analyzed for blood glucose by oxidase-peroxidase method using Accu-Chek Sensor Comfort (Roche Diagnostics, Germany) test strips.

### 2.12. Statistical Analysis

Statistical optimization was performed using Design-Expert Version 8.0.6.1 software (Stat-Ease Inc., USA). The *in vivo *data were tested for significant differences (*P* < 0.05) by paired samples *t*-test. All other data was analyzed with simple statistics. The simple statistical analysis and paired samples *t*-test were conducted using MedCalc software, version 11.6.1.0.

## 3. Results and Discussion

IHM was isolated from ispaghula (*Plantago ovata* F.) husk, and the average yield of IHM was found 39.86% w/w. 

For the 3^2^ factorial design, a total of 9 trial formulations were proposed by Design-Expert Version 8.0.6.1 software. According to this trial proposal, various glibenclamide-loaded IHM-alginate beads were prepared by ionotropic-gelation technique. When various dispersion mixtures containing polymer-blend (SA and IHM) and glibenclamide were dropped into the solutions containing calcium ions, gelled glibenclamide-loaded IHM-alginate beads were formed instantaneously due to electrostatic interaction between negatively charged alginate ions and positively charged calcium ions present in the cross-linking solutions. 

Overview of matrix of the design including investigated responses (DEE and *R*_10 h_) was presented in Table 1. The values of DEE and *R*_10 h_, measured for all trial formulations, were fitted in the 3^2^ factorial design to get model equations. The Design-Expert Version 8.0.6.1 software provided quadratic model equations involving individual main factors and interaction factors for all response parameters. The results of the ANOVA indicated that these models were significant for all response parameters (Table 2). 

The model equation relating DEE  (%) as response became DEE  (%) = 87.24−  5.22*X*_1_−  2.49*X*_2_−  0.06*X*_1_ *X*_2_+  0.41*X*_1_^2^+  0.35*X*_2_^2^ (*R* ^2^ = 0.9999; *F* value = 10398.24; *P* < 0.05). 

The model equation relating *R*_10 h_(%) as response became *R*_10 h_  (%) = 83.94+  2.37*X*_1_+  0.01*X*_2_+  0.34*X*_1_ *X*_2_−  0.13*X*_1_^2^ − 0.23*X*_2_^2^  (*R* ^2^ = 0.9981; *F* value = 309.07; *P* < 0.05). 

Model simplification was carried out by eliminating nonsignificant terms (*P* > 0.05) from previously mentioned model equations [23], giving DEE  (%) = 87.24 − 5.22*X*_1_ − 2.49*X*_2_ − 0.06*X*_1_ *X*_2_ + 0.41*X*_1_^2^+  0.35*X*_2_^2^ and *R*_10 h_(%) = 83.94 + 2.37*X*_1_ + 0.01*X*_2_ + 0.34*X*_1_ *X*_2_ − 0.23*X*_2_^2^.

**Figure 1:**
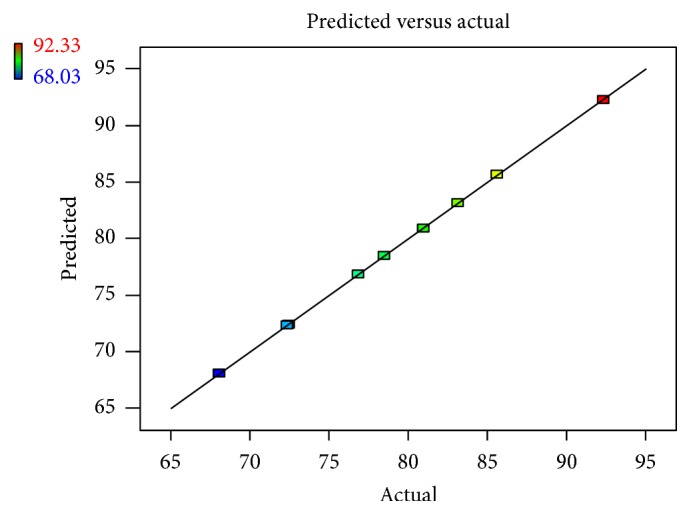
Linear correlation plot relating DEE  (%) between the actual and the predicted values.

**Figure 2:**
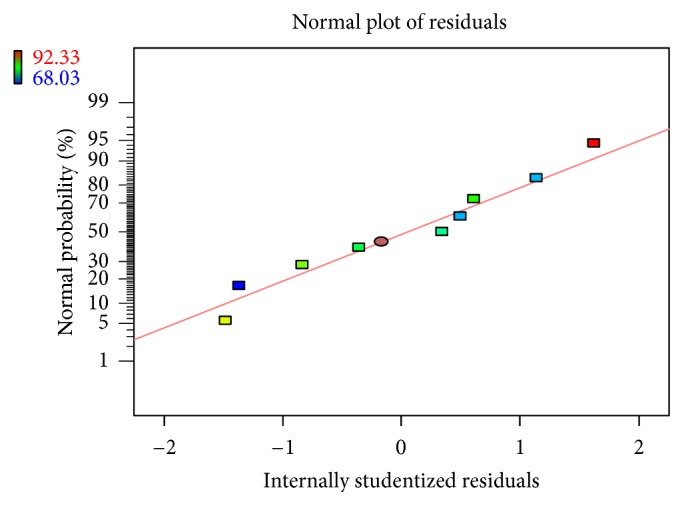
Normal residual plot relating DEE  (%) showing the scatter of the residuals versus predicted values.

**Figure 3:**
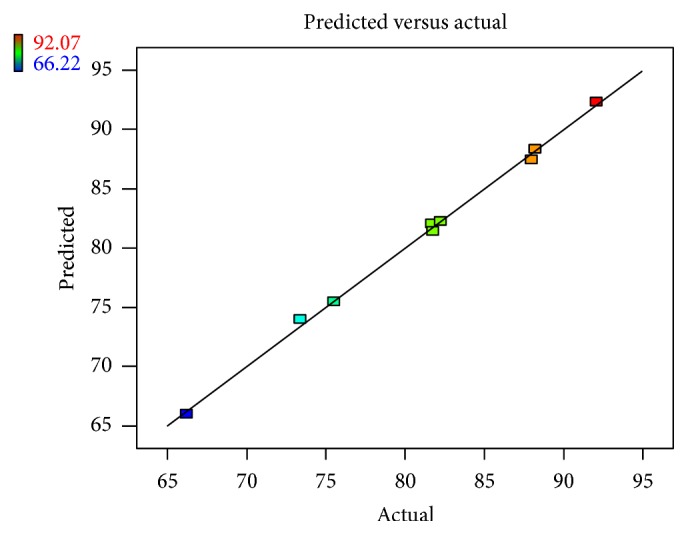
Linear correlation plot relating *R*_10 h_(%) between the actual and the predicted values.

**Figure 4:**
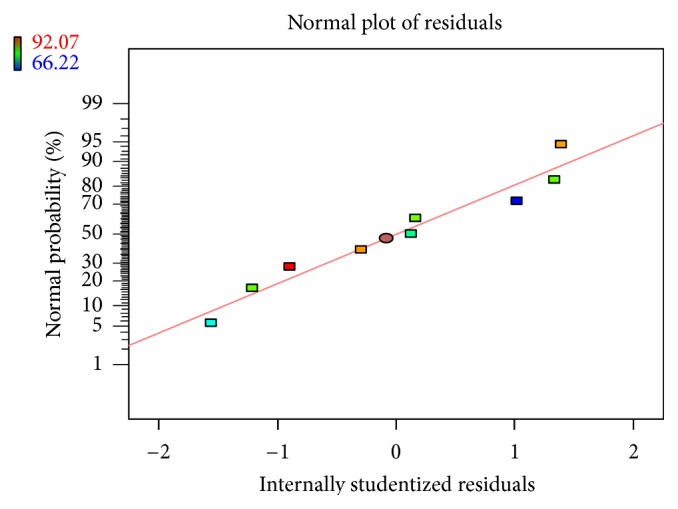
Normal residual plot relating *R*_10 h_(%) showing the scatter of the residuals versus predicted values.

**Figure 5:**
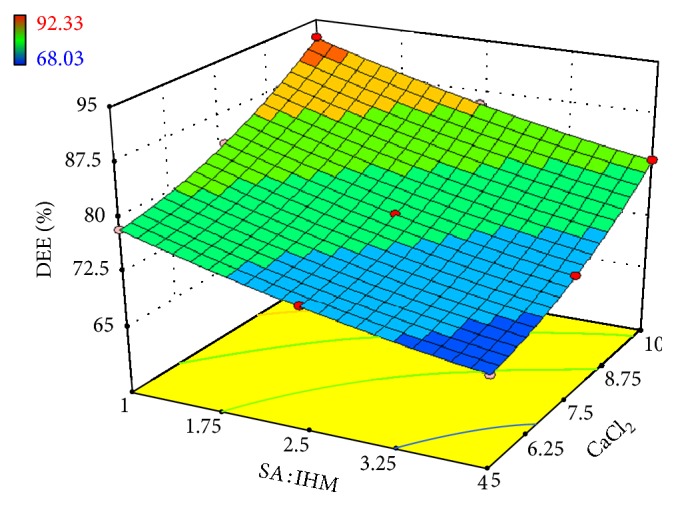
Effect of SA: IHM and concentration of CaCl_2_ on DEE presented by response surface plot.

**Figure 6:**
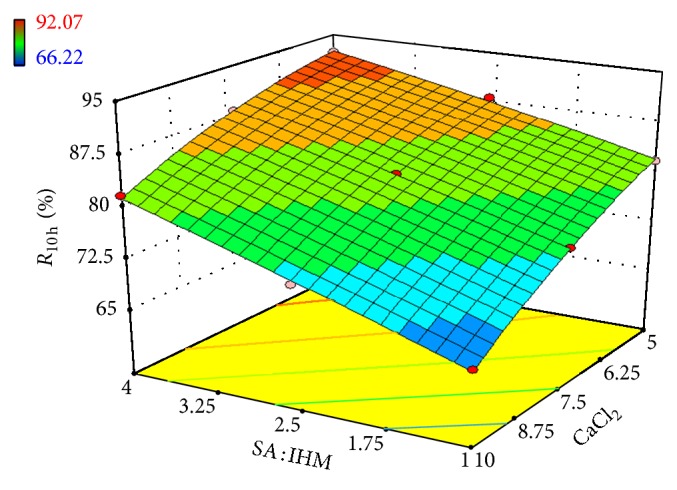
Effect of SA: IHM and concentration of CaCl_2_ on *R*_10 h_ presented by response surface plot.

**Figure 7:**
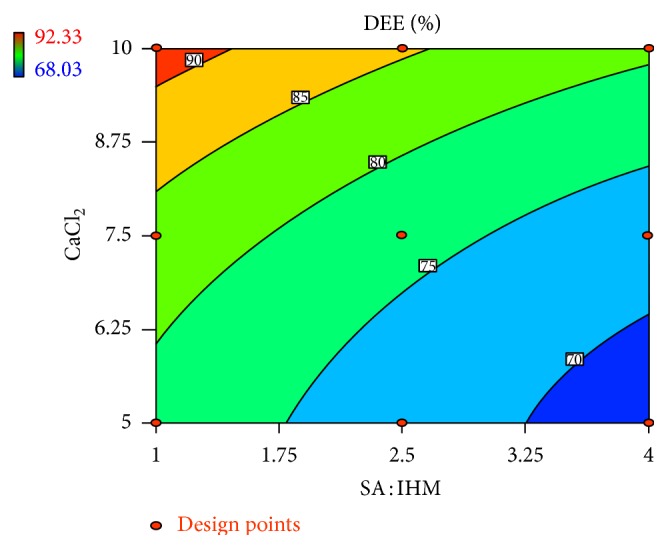
Effect of SA: IHM and concentration of CaCl_2_ on DEE presented by contour plot.

**Figure 8:**
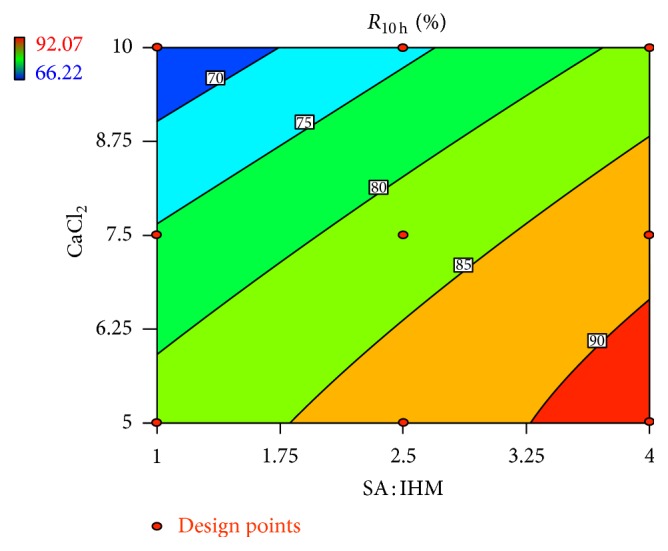
Effect of SA: IHM and concentration of CaCl_2_ on *R*_10 h_ presented by contour plot.

Linear correlation plots between the actual, the predicted response variables are presented in Figures 1 and 2, and their corresponding residual plots showing the scatter of the residuals versus predicted values are presented in Figures 3 and 4. The influences of main effects (factors) on responses (here, DEE and *R*_10 h_) were further elucidated by response surface methodology. Response surface methodology is a widely proficient approach in the development and optimization of drug delivery devices [5, 8, 28]. Response surface methodology encompasses the generation of model equations of the investigated responses over the experimental domain to determine optimum formulation (*s*) [29]. The three-dimensional response surface plot is very useful in learning about the main and interaction effects of the independent variables (factors), whereas two-dimensional contour plot gives a visual representation of values of the response [5]. The three-dimensional response surface plot relating DEE (Figure 5) indicates the increment of DEE with the lowering of SA: IHM (*X*_1_) and increasing of CaCl_2_ concentration (*X*_2_). However, an increment in *R*_10 h_ values with the increasing of SA: IHM (*X*_1_) and lowering of CaCl_2_ concentration (*X*_2_) is indicated by the three-dimensional response surface plot relating *R*_10 h_ (Figure 6). All the two-dimensional contour plots relating measured responses (Figures 7 and 8) showed nonlinear relationships between independable variables, investigated for this study. 

**Figure 9:**
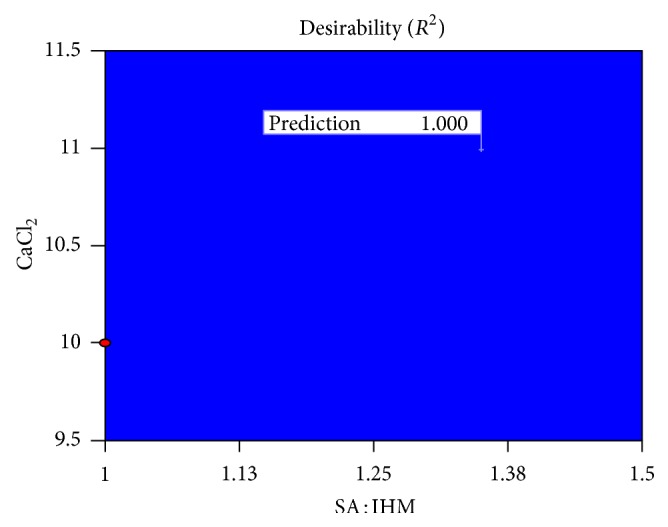
The desirability plot indicating desirable regression ranges to get optimal formula.

**Figure 10:**
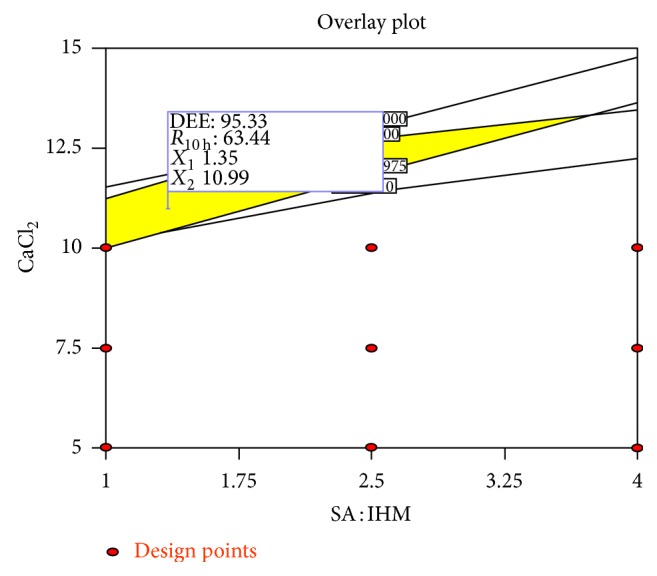
The overlay plot indicating the region of optimal process variable settings.

Numerical optimization technique using the desirability approach was employed to develop optimized formulations with desired response (optimum quality). The desirable ranges of the independable variables (factors) were restricted to 1.00 ≤ *X*_1_ ≤ 1.50 and 9.50 ≤ *X*_2_ ≤ 11.50, whereas the desirable ranges of responses were restricted to 95.00 ≤ DEE ≤ 100.00% and 60.00 ≤ *R*_10 h_ ≤ 65.00%. The optimal values of responses were obtained by numerical analysis using the Design-Expert Version 8.0.6.1 software based on the criterion of desirability. The desirability plot indicating desirable regression ranges for optimal process variable settings was presented in Figure 9, and overlay plot indicating the region of optimal process variable settings was presented in Figure 10. In order to evaluate the optimization capability of these models generated according to the results of 3^2^ factorial design, optimized glibenclamide-loaded IHM-alginate beads were prepared using one of the optimal process variable settings proposed by the design (prediction *R* ^2^ = 1). The selected optimal process variable setting used for the formulation of optimized formulation was *X*_1_ = 1.35 and *X*_2_ = 10.99. The optimized beads containing glibenclamide (F-O) were evaluated for DEE  (%) and *R*_10 h_(%).  Table 3 lists the results of experiments with predicted responses by the mathematical models and those actually observed. The optimized glibenclamide-loaded IHM-alginate beads (F-O) showed DEE of 94.43 ± 4.80% and *R*_10 h_ of 65.78 ± 3.44% with small error values (0.94, and −3.69, resp.), indicating that mathematical models obtained from the 3^2^ factorial design were fitted well. 

The DEE  (%) of all these glibenclamide-loaded IHM-alginate beads was within the range between 68.03 ± 1.77 and 94.43 ± 4.80% w/w (Tables 1 and 3). It was observed that DEE  (%) was increased with the lowering of SA: IHM in polymer-blend, which may be due to increase in viscosity of the polymeric solution by the IHM addition as polymeric-blend with SA. This might have prevented drug leaching to the cross-linking solution and the elevation of cross-linking by CaCl_2_. Again, the DEE of these beads was increased with increasing CaCl_2_ concentration in cross-linking solutions, due to the high degree of cross-linking by the concentrated calcium ions. The glibenclamide-loaded IHM-alginate beads prepared using lower CaCl_2_ concentration might have larger pores due to insufficient cross-linking, and drug leaching may occur through the pores that may result in lower drug encapsulation [17].

The average bead size of glibenclamide-loaded IHM-alginate beads was within the range of 0.80 ± 0.06 to 1.47 ± 0.12 mm (Table 4). Increase in the average size of beads was found with the increasing incorporation of IHM as a polymer-blend with SA. This could be attributed due to the increase in viscosity of polymer-blend solution with incorporation of IHM in increasing ratio that in turn increased the droplet size of polymer-blend solutions to the cross-linking solutions during preparation. Again, the decrease in average size of these formulated IHM-alginate beads was observed, when concentrated CaCl_2_ solution was used for cross-linking, which might be due to shrinkage of polymeric gel by higher degree of cross-linking. 

**Figure 11:**
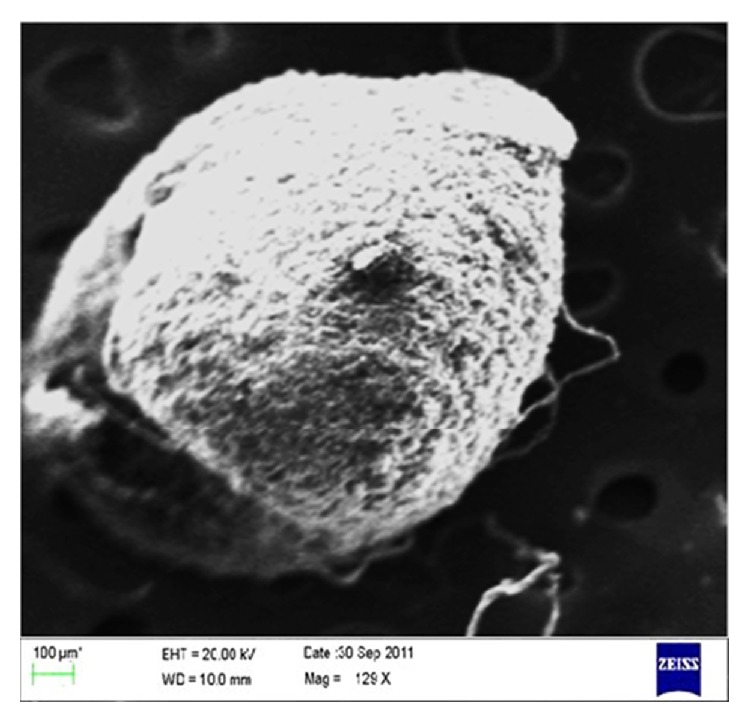
Scanning electron microphotograph of the surface of optimized glibenclamide-loaded IHM-alginate beads (F-O).

The surface morphological analysis of glibenclamide-loaded IHM-alginate beads was visualized by SEM and presented in Figure 11. The SEM photograph of these beads possessed irregular shape without forming agglomeration. Their surface morphologies appeared to have rough with characteristic large wrinkles and cracks, as it was evident from the SEM photographs. These cracks and wrinkles might be caused by partly collapsing the polymeric gel network during drying. 

**Figure 12:**
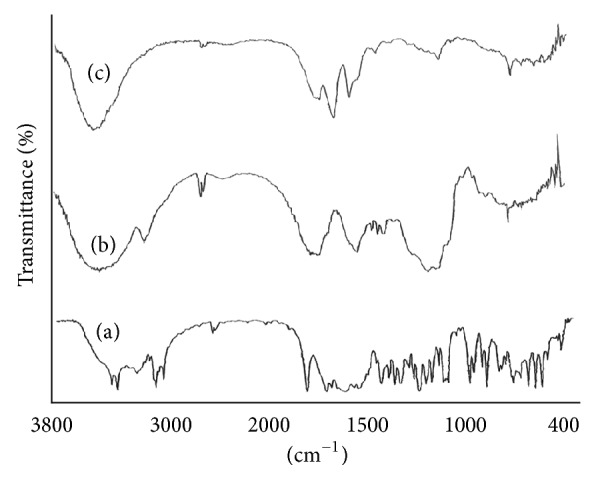
The FTIR spectra of pure glibenclamide (a), optimized glibenclamide-loaded IHM-alginate beads (b), and glibenclamide-loaded IHM-alginate beads without drug (c).

The FTIR spectra of pure glibenclamide, glibenclamide-loaded IHM-alginate beads, and IHM-alginate beads without drug are shown in Figure 12. The FTIR spectrum of pure glibenclamide and the principal absorption peaks appeared at 3314 cm^−1^ due to the –NH stretching, 3116 cm^−1^ for aromatic hydrogen absorption, and a peak at 1717 cm^−1^ occurs due to –C=O absorption peak. In the FTIR spectrum of glibenclamide-loaded IHM-alginate beads, various characteristic peaks of glibenclamide appeared without any significant shifting. This indicates that glibenclamide maintained its identity after formulation of IHM-alginate beads through ionotropic-gelation technique. In both the FTIR spectra of glibenclamide-loaded IHM-alginate beads and IHM-alginate beads without drug, the strong and broad absorption band peaks had been observed between 3600–3200 cm^−1^ due to –OH stretching along with some complex bands in the region 1200–1030 cm^−1^ due to –C–O and C–O–C stretching vibrations, which are the characteristic of the natural polysaccharides. In addition, absorption bands in the regions 930–820 cm^−1^ and 785–730 cm^−1^ were also observed due to vibrational modes of pyranose rings of polysaccharides. The presence of strong asymmetric stretching absorption band between 1650 cm^−1^ and 1620 cm^−1^ and weaker symmetric stretching band near 1420 cm^−1^ supported the presence carboxylate anion of alginate structure. The FTIR analysis confirmed the compatibility of the glibenclamide with SA and IHM used to prepare the glibenclamide-loaded IHM-alginate beads by ionotropic-gelation technique.

**Figure 13:**
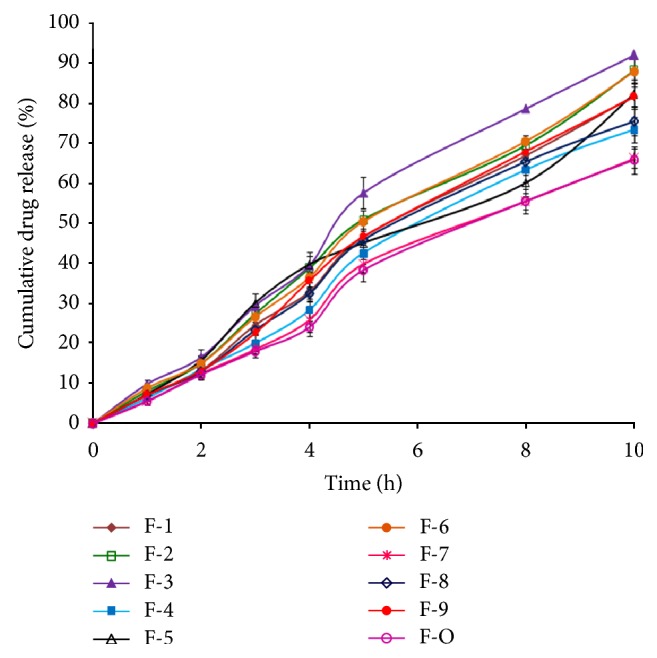
*In vitro *glibenclamide release from various IHM-alginate beads (mean ± S.D., *n* = 3).

The *in vitro* glibenclamide release studies were carried out for glibenclamide-loaded IHM-alginate beads in the 0.1 N HCl (pH, 1.2) for first 2 hours and then in phosphate buffer (pH, 7.4) for next 8 hours. All these beads showed prolonged glibenclamide release over 10 hours (Figure 13). Glibenclamide release from these IHM-alginate beads in the acidic pH was found slow due to the shrinkage of alginate at acidic pH. The trace amount of drug release at the initial stage of the dissolution study could probably be due to the surface adhered drug. After that, glibenclamide release was observed faster in phosphate buffer (pH, 7.4) comparatively, due to the higher swelling rate of these beads in phosphate buffer. The cumulative drug released from these formulated beads containing glibenclamide in 10 hours (*R*_10 h_, %) was within the range of 65.78 ± 3.44% to 92.07 ± 4.05%, and this was found to be higher with the decreasing SA to IHM ratio in the polymer-blend and increasing CaCl_2_ concentration in cross-linking solution. In case of comparatively higher IHM containing beads, the more hydrophilic property of IHM could bond better with water to form viscous gel-structure. This might blockade the pores on the surface of beads and sustain drug release profile. Again, the glibenclamide release from IHM-alginate beads prepared using higher CaCl_2_ concentration was comparatively sustained than the beads formulated with that of lower concentration. The higher concentration of CaCl_2_ (cross-linker) could produce high degree of cross-linking and thereby slower the drug release from highly cross-linked glibenclamide-loaded IHM-alginate beads. 

The *in vitro *drug release data from various glibenclamide-loaded IHM-alginate beads were evaluated kinetically using various mathematical models like zero-order, first-order, Higuchi, and Korsmeyer-Peppas models. The accuracy and prediction ability of these models was determined using regression analysis. The result of the curve fitting (*R* ^2^) into various mathematical models is given in Table 5. When the respective *R* ^2^ of glibenclamide-loaded IHM-alginate beads was compared, it was found to follow the zero-order model (*R* ^2^ = 0.992 to 0.997) over a period of 10 hours. This was also observed to be closest to Korsmeyer-Peppas model (*R* ^2^ = 0.985 to 0.994). The best fit of zero-order model indicated that the glibenclamide release from these IHM-alginate beads followed controlled-release pattern. The values of diffusional exponent (*n*) determined from Korsmeyer-Peppas model ranged from 1.025 to 1.115, indicating the drug release from these glibenclamide-loaded IHM-alginate beads following the super case-II transport mechanism controlled by swelling and relaxation of polymeric-blend (SA-IHM) matrix. This could be attributed due to polymer dissolution and polymeric chain enlargement or relaxation. 

**Figure 14:**
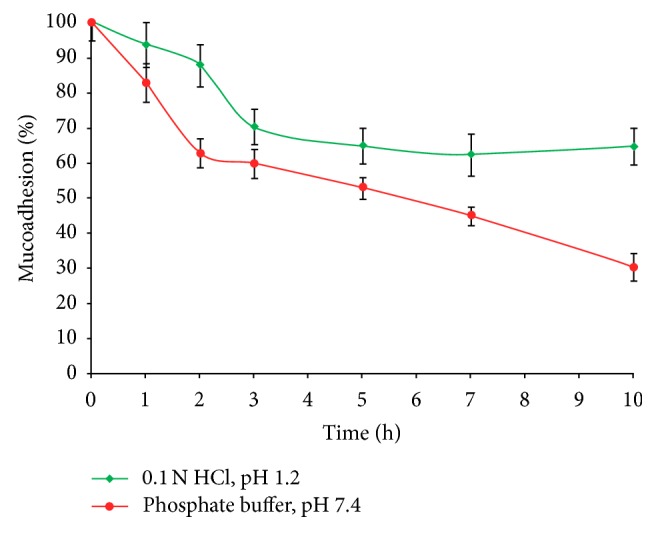
Result of *ex vivo* wash-off test to assess mucoadhesive properties of optimized glibenclamide-loaded IHM-alginate beads in 0.1 N HCl, pH 1.2, and phosphate buffer, pH 7.4 (mean ± S.D., *n* = 3).

The *ex vivo *wash-off behavior of optimized glibenclamide-loaded IHM-alginate beads (F-O) using goat intestinal mucosa was found faster in intestinal pH (7.4) than that in gastric pH (1.2). In gastric pH, the percentage of beads adhered onto the goat intestinal mucosal tissue varied from 64.88 ± 5.06% over 10 hours, whereas this was 30.47 ± 3.86% in intestinal pH (Figure 14). Thus, the results of the *ex vivo *wash-off test indicated that the newly developed optimized glibenclamide-loaded IHM-alginate beads had good mucoadhesivity. 

**Figure 15:**
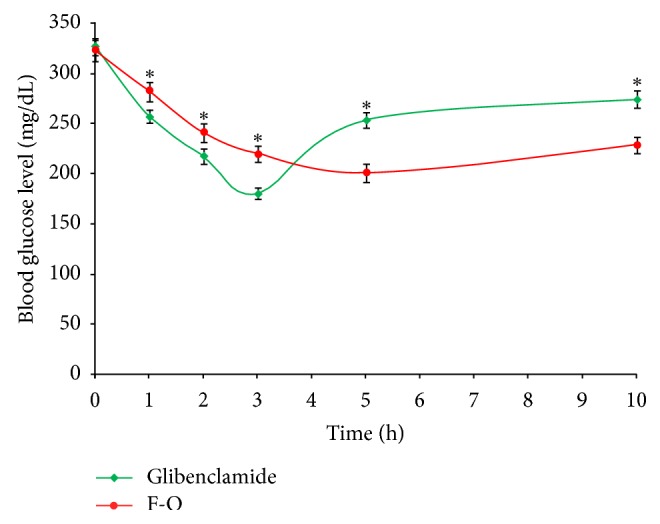
Comparative *in vivo *blood glucose level in alloxan-induced diabetic rats after oral administration of pure glibenclamide and optimized glibenclamide-loaded IHM-alginate mucoadhesive beads (mean ± S.D., *n* = 6).

**Figure 16:**
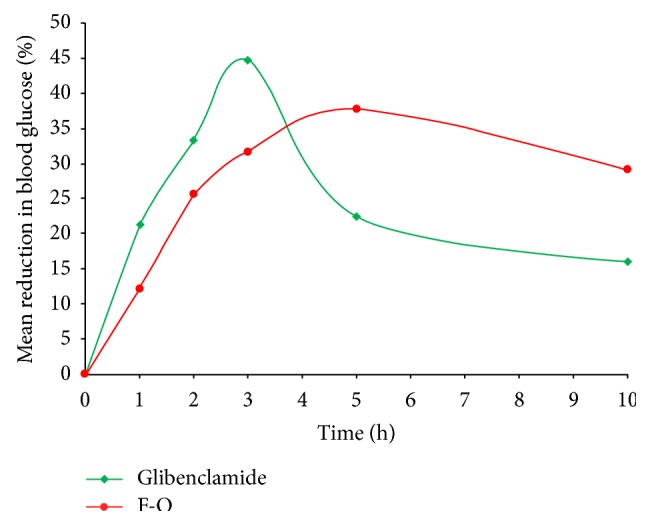
Comparative *in vivo* mean percentage reduction in blood glucose level in alloxan-induced diabetic rats after oral administration of pure glibenclamide and optimized glibenclamide-loaded IHM-alginate mucoadhesive beads (F-O).

In alloxan-induced diabetic rats, the comparative *in vivo *blood glucose level and the mean percentage reduction in blood glucose level after oral administration of pure glibenclamide and optimized glibenclamide-loaded IHM-alginate mucoadhesive beads (F-O) are presented in Figures 15 and 16, respectively. In case of the group treated with pure glibenclamide (Group A), a rapid reduction in blood glucose level was observed within 2-3 hours of administration, and after that, the blood glucose level recovered rapidly towards the normal level. In case of the group (Group B) treated with optimized glibenclamide-loaded IHM-alginate mucoadhesive beads, the reduction in blood glucose level was found slower than that of the group treated with pure glibenclamide (Group A) up to 3 hours. Significant differences (*P* < 0.05) were found between the blood glucose levels after administration of pure glibenclamide and optimized glibenclamide-loaded IHM-alginate mucoadhesive beads (F-O) at each time point measured. However, the reductions in glucose level were increased gradually with the increment of time in case of Group B (treated with optimized glibenclamide-loaded IHM-alginate mucoadhesive beads) and were sustained over 10 hours. A 25% reduction in glucose level is considered a significant hypoglycemic effect [29]. Therefore, the significant hypoglycemic effect by the optimized glibenclamide-loaded IHM-alginate mucoadhesive beads (F-O) was observed over 10 hours. 

## 4. Conclusion

In this investigation, glibenclamide-loaded IHM-alginate mucoadhesive beads were successfully developed and optimized. These developed optimized mucoadhesive beads demonstrated high drug encapsulation, good mucoadhesivity with the biological membrane, sustained drug release profile at a controlled rate, and significant antidiabetic activity in alloxan-induced diabetic rats over prolonged period after oral administration. Therefore, these glibenclamide-loaded IHM-alginate mucoadhesive beads were found suitable for prolonged systemic absorption of glibenclamide through sustained drug release and mucoadhesive properties after oral administration maintaining tight blood glucose level and improved patient compliance in the management of non-insulin-dependent diabetes mellitus. Moreover, the technique for the preparation of these beads was found simple, economical, and consistent. This type of beads can also be exploited for drug delivery of other drugs to improve their bioavailability and therapeutic efficacy.

## Figures and Tables

**Table 1:** Experimental plan of 3^2^ factorial design (coded values in bracket) with observed response values for different glibenclamide-loaded IHM-alginate beads.

Batch code	Normalized levels of factors employed		Responses	
	SA : IHM	CaCl_2 _concentration	DEE (%)∗	*R*_10 h_ (%)∗
F-1	4 (+1)	10.00 (+1)	80.90 ± 3.02	81.76 ± 3.15
F-2	4 (+1)	7.50 (0)	72.31 ± 2.79	88.23 ± 3.17
F-3	4 (+1)	5.00 (−1)	68.03 ± 2.32	92.07 ± 4.05
F-4	2.50 (0)	10.00 (+1)	85.56 ± 3.45	73.37 ± 3.22
F-5	2.50 (0)	7.50 (0)	76.82 ± 3.48	82.27 ± 3.46
F-6	2.50 (0)	5.00 (−1)	72.44 ± 2.87	88.00 ± 3.84
F-7	1.00 (−1)	10.00 (+1)	92.33 ± 3.82	66.22 ± 2.51
F-8	1.00 (−1)	7.50 (0)	83.12 ± 3.57	75.52 ± 3.58
F-9	1.00 (−1)	5.00 (−1)	78.49 ± 3.02	81.70 ± 2.33

^*^Mean ± S.D.; n = 3.

**Table 2:** Summary of ANOVA for response surface quadratic models.

Source	Sum of squares	df∗	Mean square	*F* value	*P* value Prob > *F*
For DEE (%)					

Model	454.39	5	90.88	10398.24	<0.0001 (S)
*X*_1_	178.00	1	178.00	20366.23	<0.0001 (S)
*X*_2_	264.67	1	264.67	30283.30	<0.0001 (S)
*X*_1_ *X*_2_	0.23	1	0.23	25.82	0.0147 (S)
*X*_1_^2^	1.72	1	1.72	196.51	0.0008 (S)
*X*_2_^2^	9.78	1	9.78	1119.35	<0.0001 (S)

For *R*_10 h_ (%)					

Model	532.16	5	106.43	309.07	0.0003 (S)
*X*_1_	248.58	1	248.58	721.87	0.0001 (S)
*X*_2_	272.30	1	272.30	790.73	<0.0001 (S)
*X*_1_ *X*_2_	6.68	1	6.68	19.40	0.0217 (S)
*X*_1_^2^	0.18	1	0.18	0.45	0.5263 (NS)
*X*_2_^2^	4.42	1	4.42	12.84	0.0372 (NS)

^*^df indicates degree of freedom.

S and NS indicate significant and not significant, respectively.

**Table 3:** Results of experiments for confirming optimization capability.

Batch code	SA : IHM	CaCl_2_ concentration	DEE (%)∗	*R*_10 h_ (%)∗
F-O	1.35	10.99	Actual values	
			94.43 ± 4.80	65.78 ± 3.44
			Predicted values	
			95.33	63.44

	% Error^#^		0.94	−3.69

^*^mean ± S.D.; *n* = 3.

^#^percentage of error (%) = (actual value − predicted value)/predicted value × 100.

**Table 4:** Mean diameter of IHM-alginate beads containing glibenclamide measured by optical microscopic method.

Batch codes	Mean diameter (mm)^#^
F-1	0.92 ± 0.07
F-2	0.99 ± 0.10
F-3	1.47 ± 0.12
F-4	0.88 ± 0.10
F-5	0.93 ± 0.08
F-6	1.22 ± 0.14
F-7	0.84 ± 0.08
F-8	0.89 ± 0.11
F-9	1.04 ± 0.12
F-O	0.80 ± 0.06

^#^Mean ± S.D., *n* = 20.

**Table 5:** Results of curve fitting of the *in vitro* glibenclamide release data from different IHM-alginate mucoadhesive beads.

Formulation codes	Correlation coefficient (*R* ^2^)				Release exponent (*n*)
	Zero-order	First-order	Higuchi	Korsmeyer-Peppas	
F-1	0.997	0.900	0.552	0.992	1.094
F-2	0.997	0.892	0.590	0.994	1.055
F-3	0.996	0.902	0.607	0.991	1.025
F-4	0.995	0.915	0.533	0.994	1.103
F-5	0.992	0.832	0.599	0.985	1.084
F-6	0.998	0.910	0.586	0.991	1.037
F-7	0.996	0.912	0.527	0.992	1.115
F-8	0.986	0.898	0.560	0.992	1.086
F-9	0.998	0.906	0.548	0.990	1.100
F-O	0.997	0.902	0.530	0.994	1.108
